# Supplementary material for: The Expression of Cell Cycle-Related Genes in USP8-Mutated Corticotroph Neuroendocrine Pituitary Tumors and Their Possible Role in Cell Cycle-Targeting Treatment
Source: Cancers (Basel). 2022 Nov 14;14(22):5594. doi: 10.3390/cancers14225594 (PMC9688166; doi:10.3390/cancers14225594)
Supplement: Supplementary file 1 [file cancers-14-05594-s001.zip › cancers-1953458-supplementary/Figure S1.pdf]

Original western blots used for manuscript Figure 4

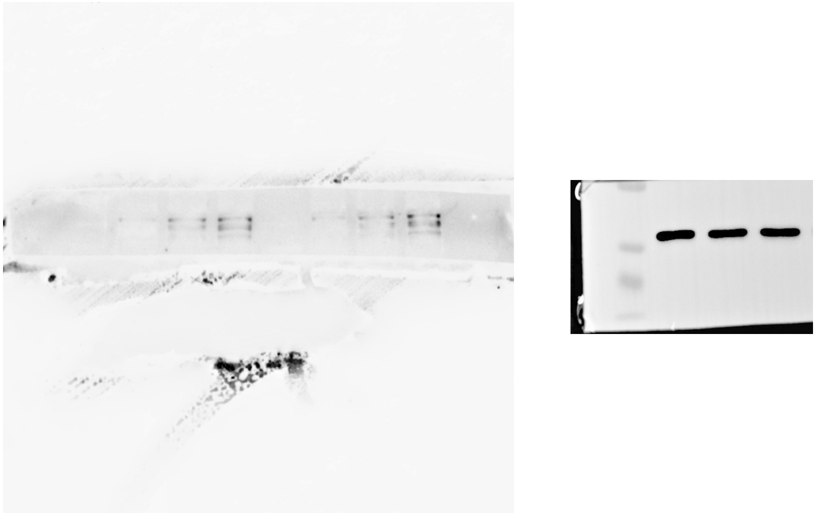

Original western blots used for manuscript Figure 5

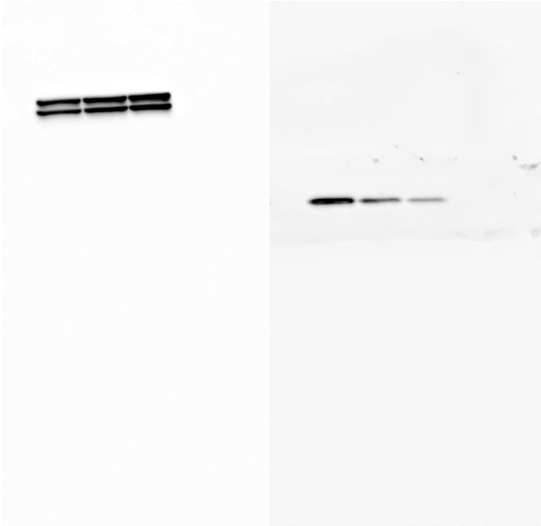

Original western blots used for manuscript Figure 6

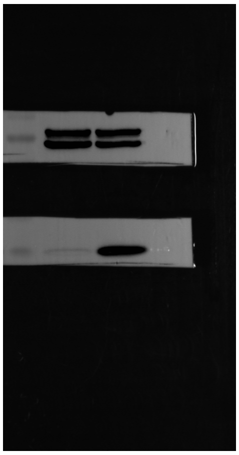

Figure S1. Original western blot figures used for manuscript figures
